# Chromatin Organization in Sperm May Be the Major Functional Consequence of Base Composition Variation in the Human Genome

**DOI:** 10.1371/journal.pgen.1002036

**Published:** 2011-04-07

**Authors:** Tanya Vavouri, Ben Lehner

**Affiliations:** 1 EMBL-CRG Systems Biology Unit, Centre for Genomic Regulation, Universitat Pompeu Fabra, Barcelona, Spain; 2 Institució Catalana de Recerca i Estudis Avançats (ICREA), Centre for Genomic Regulation, Universitat Pompeu Fabra, Barcelona, Spain; The University of North Carolina at Chapel Hill, United States of America

## Abstract

Chromatin in sperm is different from that in other cells, with most of the genome packaged by protamines not nucleosomes. Nucleosomes are, however, retained at some genomic sites, where they have the potential to transmit paternal epigenetic information. It is not understood how this retention is specified. Here we show that base composition is the major determinant of nucleosome retention in human sperm, predicting retention very well in both genic and non-genic regions of the genome. The retention of nucleosomes at GC-rich sequences with high intrinsic nucleosome affinity accounts for the previously reported retention at transcription start sites and at genes that regulate development. It also means that nucleosomes are retained at the start sites of most housekeeping genes. We also report a striking link between the retention of nucleosomes in sperm and the establishment of DNA methylation-free regions in the early embryo. Taken together, this suggests that paternal nucleosome transmission may facilitate robust gene regulation in the early embryo. We propose that chromatin organization in the male germline, rather than in somatic cells, is the major functional consequence of fine-scale base composition variation in the human genome. The selective pressure driving base composition evolution in mammals could, therefore, be the need to transmit paternal epigenetic information to the zygote.

## Author Summary

In most cells, DNA is packaged by protein complexes called nucleosomes. In sperm, however, nucleosomes are only retained at a small fraction of the genome, particularly at the start sites of genes. In this work, we show that the sites at which nucleosomes are retained in sperm are specified by variation in the base composition of the human genome. At a fine scale, the human genome varies extensively in the content of GC versus AT base pairs, and we find that in both genic and non-genic regions this predicts very well where nucleosomes are retained in mature sperm. These regions include transcription start sites, especially for genes that are expressed in all cells and for genes that regulate development. We also report that regions that retain nucleosomes in sperm are likely to be protected from DNA methylation in the early embryo, suggesting a further connection between the presence of nucleosomes on the paternal genome and the establishment of gene regulation in the embryo. Based on these results, we propose that an important selective pressure on base composition evolution in mammalian genomes may be the requirement to organize chromatin in sperm in a way that facilitates gene regulation in the early embryo.

## Introduction

The chromatin of mature sperm differs dramatically from that of other cell types. Most of the sperm genome is packaged by small basic proteins called protamines, with only a few genomic sites remaining bound by nucleosomes [1], [2], [3], [4], [5]. This change in DNA packaging takes place towards the end of male germline development in transcriptionally inactive spermatids and results in a highly compact genome that fits in the small volume of the sperm head [2], [6]. In contrast to the nucleosome structure that consists of ∼147 bp of DNA wrapped around a histone octamer, individual protamine molecules bind one turn of the DNA helix [7]. In mature sperm, protamines compact the genome into large doughnut-shaped toroids, each containing ∼50 kbp of the haploid genome [2], [8], [9]. This compact packaging of the sperm genome is essential for fertility, genome integrity, and early embryonic development [7], [10], [11], [12].

In human sperm about 4% of the genome remains bound by nucleosomes [5]. Sites of nucleosome retention are dispersed along chromosomes but are not random. Instead, they are strikingly consistent among individuals [3], [4], [13]. Nucleosome retention sites are also enriched in particular genomic regions [13], [14], [15], and recent genome-wide localization analyses have reported that nucleosomes are preferentially retained in gene promoters and at loci that regulate development [3], [5]. However, despite these genome-wide maps, the signals that specify retention sites are unknown.

Although they are transcriptionally inactive, mature spermatozoa do contain nucleosomes containing histones marked by post-translational modifications, including both activation (e.g. methylation of histone H3 lysine 4) and repression marks (e.g. tri-methylation of H3 at lysine 27) [3], [4], [5]. Interestingly, both paternal nucleosomes [16] and histone modifications [17] are transmitted to the early zygote, and so have the potential to propagate paternal epigenetic information to the early embryo [18]. It is thus of great interest to understand how sites of nucleosome retention in sperm are determined, as these sites specify where epigenetic information transfer can potentially occur from the paternal germline to the zygote [19], [20], [21].

In somatic cells and in lower eukaryotes several important influences on nucleosome occupancy and positioning have been demonstrated. First, many nucleosomes are located ‘statistically’ [22] relative to nucleosomes positioned by transcription and other DNA-binding proteins [23], [24], [25]. Second, nucleosomes do not bind to all DNA-sequences with equal affinity. Rather, they have clear binding preferences that can be quantified *in vitro* [25], [26], [27] and predicted using sequence-based binding models [28], [29], [30], [31], [32].

*In vitro*, nucleosomes bind preferentially to GC-rich DNA [31], [32]. GC-rich sequences have increased flexibility that may help the wrapping of DNA around the histone octamer. Further, poly(dA∶dT) motifs destabilize the formation of nucleosomes [30], [31]. Indeed it has long been speculated that fine-scale base composition variation in mammalian genomes may relate to chromatin structure [33]. Many transcription start sites and regulatory regions are GC-rich and are predicted to have high intrinsic nucleosome affinity [34]. However, *in vivo* analysis of a number of mouse GC-rich promoters reached the opposite conclusion: GC-rich promoters were depleted of nucleosomes *in vivo* [35]. Thus, although GC-rich sequences have high intrinsic binding specificity for nucleosomes, in somatic cells other processes such as transcription may have a more important influence on nucleosome occupancy over functionally important regions of the genome.

GC-content peaks are found at the promoters of many human genes, where they are termed CpG islands because of the elevated frequency of CpG bases [36]. High CpG-content promoters are associated with both widely expressed housekeeping genes [36], [37] and with developmental regulators such as transcription factors [38], [39]. One major epigenetic feature of CpG islands is that they tend to be largely devoid of DNA methylation [40]. CpG sites in mammalian genomes are highly methylated, but many CpG islands are established as unmethylated regions in the early embryo (although they may later gain methylation in some cases upon differentiation [41], [42]). Indeed genome-wide mapping has shown that most (but not all) CpG islands are unmethylated in human embryonic stem (ES) cells [43]. This methylation-free state, combined with the presence of transcription activation marks such as tri-methylation of histone H3 lysine 4 (H3K4me3) may maintain CpG islands accessible or ‘poised’ for transcription initiation [41]. Many CpG islands are also known to be unmethylated and associated with H3K4 methylation in sperm [4], [5], [42]. Across the genome in general, however, DNA methylation has been reported as enriched on nucleosome bound DNA [44].

In the early embryo, genome-wide erasure of DNA methylation is followed by the *de novo* establishment of methylation patterns [45]. A subset of CpG sites must therefore be protected from this non-specific methylase activity. This protection may be linked to the binding of transcriptional activators [41], [46], [47] or the presence of H3K4me3-containing nucleosomes [48]. Importantly, DNA hypo-methylation is the reason why CpG islands maintain their high CpG content: methylated CpG sites have an elevated mutation rate due to the spontaneous deamination of methylcytosine to thymine, which leads to the genome-wide depletion of CpG dinucleotides outside of unmethylated regions [42], [49].

Given the lack of transcription, we reasoned that the major influence of GC-content on chromatin organization might occur in the male germline rather than in somatic cells. Here we test this idea, and show that nucleosome retention in human sperm is indeed strikingly related to fine-scale base composition variation. Across both genic and non-genic regions of the genome, nucleosome retention sites are extremely well predicted by GC-composition. The retention of nucleosomes at GC-rich sequences with high intrinsic nucleosome affinity accounts for the previously reported enrichment of nucleosomes both at transcription start sites and at genes that regulate development. It also means that nucleosomes are retained at the start sites of most universally expressed genes, which may be important for their activation in the early embryo. Further, we report a striking association at CpG islands between nucleosome retention in sperm, and the establishment of unmethylated regions in the early embryo. This suggests that paternal nucleosome retention may assist in the establishment of these regions, possibly through the retention of H3K4me3-marked histones. Our findings suggest that chromatin organization in the male germline, rather than that in somatic cells, is the major functional consequence of fine-scale base composition variation in the human genome. We suggest that the selective pressure on this may be the requirement to propagate paternal epigenetic information to the embryo.

## Results

### Nucleosomes are retained in mature sperm at GC-rich loci

**Figure 1:**
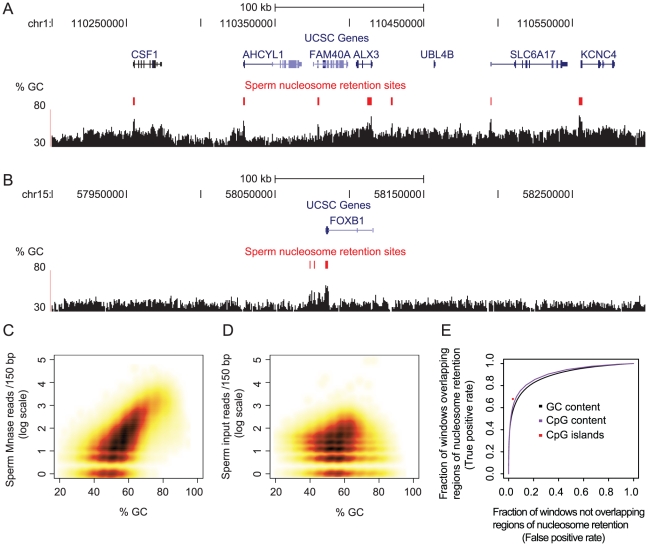
Base composition predicts sites of nucleosome retention in human sperm. Nucleosome retention sites (red) across two representative genomic regions coincide with many transcription start sites and also with local peaks of high GC-content (black). Broader retention is seen at two transcription factors that regulate development, ALX3 (A) and FOXB1 (B), and this also correlates with broader regions of high GC-content. The plots were generated using the UCSC genome browser. GC-content correlates strongly with the number of sequenced reads from mononucleosome-enriched fractions of the sperm genome (C). In comparison, there is only a very weak correlation between GC-content and the number of sequenced reads from the input genomic control (D). GC-content is an excellent predictor of regions of nucleosome retention in sperm across the human genome (E). ROC curves are shown for predictions across the genome in 150 bp windows using either GC- or CpG-content. CpG islands are also excellent predictors of sites of nucleosome retention in sperm (χ^2^ –test, p-value<2.2×10^−16^, see also Figure S6).

Sites of nucleosome retention in mature human sperm were identified genome-wide by Hammoud and co-workers using micrococcal nuclease (MNase) digestion followed by deep sequencing. Comparing mononucleosome fragments to a sonicated input control, 25,121 genomic regions were identified with statistically significant enrichment for sperm nucleosomes [5]. Mapping these regions onto the genome shows that they overlap peaks of high GC-content (Figure 1A, 1B). In genic regions, these peaks frequently occur at transcription start sites (Figure 1A) and also more broadly across some genes, particularly developmental regulators (Figure 1A, 1B).

Considering the whole genome, there is indeed a striking correlation between GC-content and the number of sequenced mononucleosome fragments isolated from sperm (Figure 1C; Pearson correlation = 0.68; p-value<2.2×10^−16^). This is not accounted for by the known GC-bias of Solexa sequencing [50] (Figure 1D, Pearson correlation = 0.12; p-value<2.2×10^−16^). Further, GC-content also correlates with nucleosome enrichment as quantified by microarray hybridization in a second study using two different extraction protocols (micrococcal nuclease digestion and salt extraction followed by restriction digestion) [3] (Figure S1).

### Base composition is an excellent predictor of nucleosome retention sites across the human genome

To formally assess the extent to which base composition predicts nucleosome retention in sperm, we divided the genome into non-overlapping 150-bp windows, and ranked these windows by their GC-content. Comparing this ranking to retention sites demonstrates that base composition alone is an excellent predictor of sperm nucleosome retention sites across the entire genome (Figure 1E). In a receiver operating characteristic (ROC) analysis, the area under the curve (AUC) is equal to 0.89. This means that for a randomly chosen pair of windows, one with retained nucleosomes and one without, there is an 89% probability of GC-content correctly classifying the two regions. Using CpG-content as a predictor provides similar performance (Figure 1E), and nucleosomes are particularly retained in annotated CpG islands (Figure 1E). As genic regions tend to be GC-rich, we then split the genome into genic and non-genic portions (excluding 1 kb around transcription start sites from the non-genic regions) and evaluated the ability of base composition to predict nucleosome retention in both fractions of the genome. Prediction was equally good in both cases, with ROC AUC = 0.89 for both the genic and non-genic portions of the genome.

### Base composition accounts for the preferential retention of nucleosomes at transcription start sites

**Figure 2:**
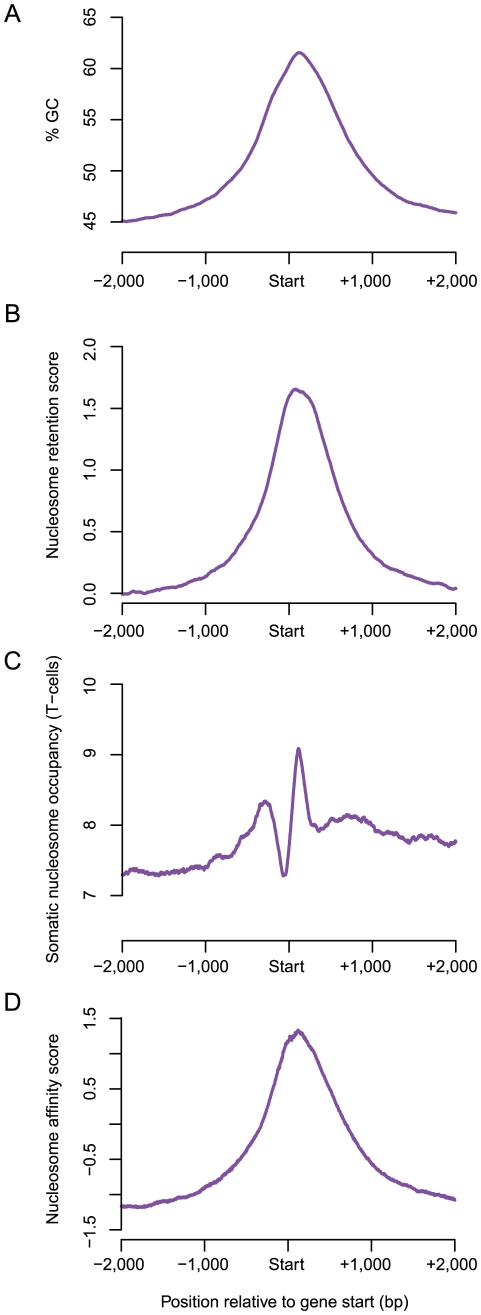
The characteristic GC-content signature of human genes account for sperm nucleosome retention at transcription start sites. Human genes show a characteristic base composition signature with high GC-content at their start sites (A), which correctly predicts high nucleosomes in sperm (B). In contrast, in a somatic tissue (resting T-cells), nucleosomes are positioned around a strong nucleosome free region at the start site, most likely due to transcription related processes (C). The high GC-content of transcription start sites means that they have high intrinsic nucleosome binding preferences (D), which correlates well with nucleosome retention in sperm, but not occupancy in somatic cells. The average plots were generated for the 4 kb region centered at the start site of all human protein-coding genes for the GC-content (A), the normalized nucleosome retention score (B), the predicted binding preferences (nucleosome model score from Kaplan *et al*) (D) and the shifted somatic nucleosome read count (C) measured in 150 bp windows.

Previously it was reported that nucleosome retention sites are enriched in gene promoters [3], [5] (see also Figure S2). More than a third of nucleosome retention regions (9,068/25,121) are located within 50 bp of a known start site (Figure S2). In contrast, only 2.9% of retention sites (718/25,121) are located at the 3′end of genes. Plotting the GC-content variation across all human genes reveals a peak at transcription start sites (Figure 2A), which closely mirrors both the nucleosome retention in sperm (Figure 2B) and the predicted *in vitro* nucleosome affinity variation (Figure 2D). In contrast, in a somatic cell (T-cell) nucleosome occupancy is not well predicted by base composition (Figure 2C), most likely because of the influence of transcription and additional DNA binding proteins [25], [51]. Thus, whereas the typical nucleosome occupancy across all genes in mature sperm is very well predicted by base composition, in somatic cells this is not the case.

### Nucleosomes are retained at the start sites of most housekeeping genes

**Figure 3:**
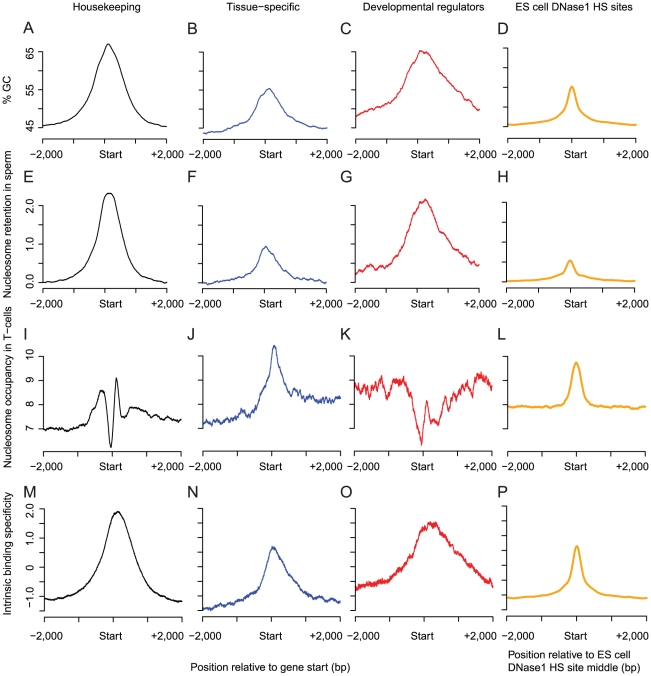
GC-content predicts variation in nucleosome retention among gene classes and at distal regulatory regions. GC-content signatures around the start sites of (A) housekeeping genes (black), (B) tissue-specific genes (genes expressed in a single tissue, blue) and (C) developmental regulators (red). Average nucleosome retention in sperm (E–G), average nucleosome occupancy in T-cells (I–K), and average nucleosome affinity around the start sites (M–O) of the same three classes of genes. Nucleosome retention in sperm, but not occupancy in T-cells, mirrors the GC-content and the intrinsic nucleosome affinity. The three gene classes contain 7,308 housekeeping genes, 1,686 tissue-specific genes and 538 transcription factors that regulate development. GC-content (D), nucleosome retention in sperm (H), nucleosome occupancy in T-cells (L), and nucleosome affinity (P) are also enriched at DNase I hypersensitive sites (HS) identified in embryonic stem (ES) cells. The average scores were calculated from 64,217 DNase I HS sites from ES cells located at least 1 kb away from any gene.

**Figure 4:**
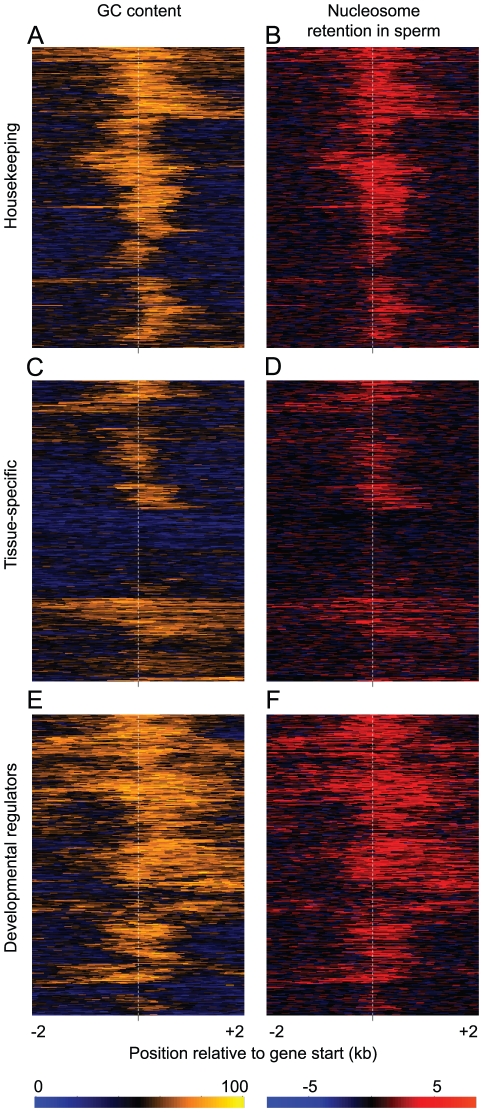
GC-content predicts sperm nucleosome retention at individual genes. GC-content (A,C,E) and sperm nucleosome retention (B,D,F) around the transcription start site of housekeeping genes (A–B), tissue-specific genes (C–D), and transcription factors that regulate development (E–F). Each row of the heat map is an individual gene. Genes are clustered according to their GC-content and the same gene ordering is used in the nucleosome retention plots. In both cases values are calculated in 150 bp windows.

Although mature sperm are transcriptionally inactive [52], it is possible that nucleosome retention relates to transcription earlier during male germline development. We compared retention at gene start sites to the transcription of genes in the male germline as quantified by deep sequencing [53]. Both highly-expressed and widely-expressed genes preferentially retain nucleosomes at their start sites (Figure S3). However, the association with expression level is largely accounted for by the association with the expression breadth of a gene (Figure S3). This is also confirmed when only considering mRNA detected in mature sperm (Figure S4). Indeed we find that 61% of ubiquitously expressed ‘housekeeping’ genes retain nucleosomes at their start sites (Figure S3B), which contrasts with only 21% of tissue-specific genes. This may relate to the need to robustly express housekeeping genes in the early embryo (see Discussion). As for the general relationship between retention sites and transcription initiation sites, this preferential nucleosome retention is accounted for by local base composition variation: housekeeping genes (Figure 3A) have higher GC content at their start sites than tissue-specific genes (housekeeping genes typically have CpG-island promoters, Figure 3B), higher nucleosome affinity (Figure 3M, 3N),and higher nucleosome retention in sperm (Figure 3E, 3F). This is not the case for somatic cells (Figure 3I, 3J), where in general base composition is a poor predictor of nucleosome occupancy at the start sites of housekeeping genes. Considering the variation in base composition and nucleosome retention in sperm within and across all individual housekeeping genes confirms these conclusions (Figure 4).

### Base composition accounts for the retention of nucleosomes at genes for transcription factors that regulate development

It was previously shown that sperm nucleosomes are also preferentially found in the promoters of genes that regulate development, particularly those encoding transcription factors such as HOX proteins [3], [5]. Indeed, 59% of genes (318/539) annotated with the Gene Ontology terms ‘DNA-dependent regulation of cellular transcription’ and ‘development’ retain nucleosomes at their start sites. Developmental transcription factors, like housekeeping genes, are also typically transcribed from CpG-island promoters. However the start sites of these developmental regulators lie within broader GC-rich regions and predicted nucleosome affinity peaks, in contrast to the sharper peaks observed at housekeeping gene starts (Figure 3C, 3O, Figure 4E). This correctly predicts the broader sperm nucleosome peak at developmental regulators (Figure 3G, Figure 4F), but not their nucleosome occupancy in somatic cells, where their start sites are generally depleted of nucleosomes (Figure 3K).

### Nucleosome retention at distal regulatory regions

In addition to gene start sites, many regulatory regions in the human genome are also GC-rich with high predicted intrinsic nucleosome affinity [34], [54]. Using DNase I hypersensitive sites in ES cells to identify putative distal regulatory regions that function in the early embryo, we find that they are also associated with nucleosome retention in sperm (Figure 3H). Thus, in addition to the promoters of developmental regulators and housekeeping genes, nucleosomes are also retained in mature sperm at distal regulatory regions that are active in the early embryo. In all cases, nucleosome retention is accounted for by local base composition variation along the human genome.

### Nucleosome retention in sperm is linked to the establishment of DNA methylation-free regions in the early embryo

**Figure 5:**
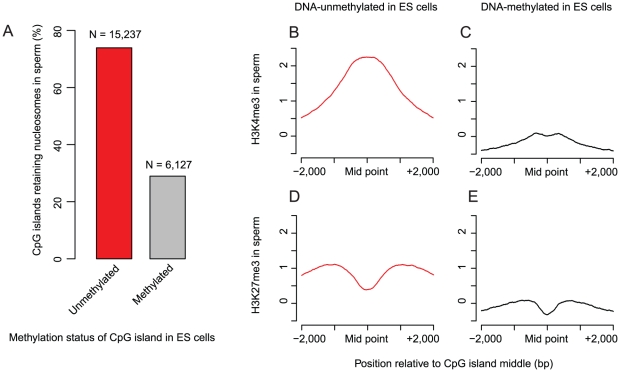
Nucleosome retention in sperm is linked to the formation of DNA methylation-free regions in the early embryo. Most (74%, 11,264/15,237) CpG islands that remain unmethylated in ES cells overlap nucleosome retention sites in sperm. In contrast, only 29% (1774/6,127) of the CpG islands that are methylated in ES cells overlap sperm nucleosome retention sites (A). CpG islands that are unmethylated in ES cells are enriched for H3K4me3 in mature sperm (B) compared to CpG islands that are methylated in ES cells (C). H3K27me3 shows moderate enrichment in sperm around CpG islands that are unmethylated in ES cells (D) compared to around CpG islands that are DNA methylated in ES cells (E).

One of the most striking epigenetic events in the early embryo is the *de novo* genome-wide re-establishment of DNA methylation at CpG sites [55]. Recently it was shown that many, but not all, CpG islands are protected from this *de novo* wave of methylation [41]. Given that nucleosomes are also enriched at many CpG islands in sperm (Figure 1E) we investigated whether these two phenomena might be linked. Strikingly, we observed a strong association between nucleosome retention at CpG islands in sperm and the establishment of unmethylated regions in the early embryo (Figure 5A). Considering all CpG islands in the genome, sperm nucleosome retention predicts the establishment of an unmethylated region with a precision of 86%, and correctly identifies 74% of all unmethylated regions. Unmethylated CpG islands in the early embryo are also strongly associated with H3K4me3 in mature sperm (Figure 5B), and to a lesser degree with H3K27me3 (Figure 5D). Thus, the retention of nucleosomes in sperm, and the modification H3K4me3, are directly or indirectly linked to the establishment of DNA methylation-free regions in the early embryo.

## Discussion

### Nucleosome retention in sperm, rather than occupancy in the soma, may be the major functional consequence of base composition variation in the human genome

**Figure 6:**
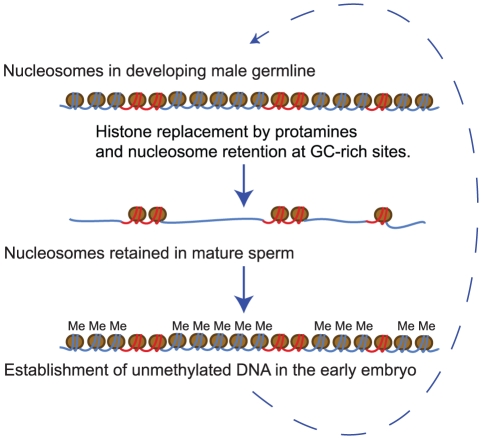
A model for nucleosome retention in human sperm. During the histone to protamine transition nucleosomes are retained at GC-rich sites which have high intrinsic affinity for nucleosomes. This results in nucleosome retention at the start sites of many genes, especially at the start sites of housekeeping genes and master regulators, as well as at distal regulatory elements. Regions that retain nucleosomes in sperm are also frequently established as free from DNA-methylation (‘Me’) in the early embryo, further suggesting a connection between the transmission of paternal nucleosomes and the establishment of gene regulation in the early embryo.

In mature sperm only a minority of the genome remains bound by nucleosomes [5]. We have shown here that nucleosome retention sites defined genome-wide in sperm by MNase digestion are strikingly predicted by the fine-scale GC-content variation along the human genome. In both genic and non-genic regions of the genome, base composition is likely to be the primary determinant of nucleosome retention (Figure 6).

High GC-content is associated with an increased binding affinity for nucleosomes *in vitro* [31], [32], which suggests that intrinsic binding preferences may account for much of the retention biases observed in sperm. It also suggests that the major consequence of intrinsic binding affinity variation along the human genome may be chromatin organization in transcriptionally quiescent sperm, rather than that in transcriptionally active somatic cells. Consistent with this, inhibiting RNA polymerase in yeast results in nucleosome occupancy that more closely matches the predicted *in vitro* binding preferences [24]. We note, however, that *in vitro* binding preferences, as quantified in a sequence-based model, are a slightly poorer genome-wide predictor of nucleosome retention in sperm than GC-content alone (data not shown). This suggests that other factors such as competition with protamines or transition proteins, CpG-binding proteins [56] or the process of DNA demethylation earlier in germline development [57] might also be important.

### Nucleosome retention may relate to the need for robust gene activation and silencing in the early embryo

The transmission of paternal nucleosomes [16] and their modifications [17] to the zygote could influence gene activity in the embryo [19], [20], [21]. For example, the inheritance of H3K4me3 and H3K27me3 at developmental loci might be important for establishing a robust silent or ‘poised’ state in the early embryo [3], [4], [5] (see also Figure S5). Consistent with this, H3K27me3 marked regions are very similar in mature sperm and in the early embryo, suggesting that this epigenetic state might be stably maintained across generations from one germline to the next [4], [5]. Similarly, we suggest that the retention of nucleosomes and activation marks (Figure S5) at housekeeping genes might be important for marking transcription start sites on the paternal genome, facilitating gene activation in the embryo.

The retention of nucleosomes at distal regulatory regions defined by DNase I hypersensitive sites is also consistent with a model in which the transmission of paternal nucleosomes and their modifications influences gene expression in the early embryo. Further, we suggest that the need to retain nucleosomes in sperm may explain why widely and highly expressed genes have high nucleosome occupancy encoded at their transcription start sites in the human genome, in contrast to the nucleosome-free regions encoded in the yeast genome [29], [58].

### Nucleosome retention in sperm is linked to the establishment of DNA methylation-free regions in the early embryo

Also consistent with a connection between nucleosome retention and gene expression in the embryos is the observation that nucleosome retention sites are established as free from DNA methylation in the early embryo. During early embryogenesis the genome-wide removal of methylation marks is followed by a wave of non-specific methylase activity [45]. Many CpG islands are protected from *de novo* methylation, and these islands are accurately predicted by their nucleosome retention in sperm (74% of regions are identified with a precision of 86%). This suggests the interesting model that paternal nucleosome inheritance might aid in the establishment of DNA methylation-free regions (Figure 6). There is evidence that the histone modification H3K4me3 can interfere with DNA methylation [48], and CpG islands that retain nucleosomes in sperm are also enriched for this mark (Figure 5). This suggests a possible mechanism for how nucleosome retention may influence DNA methylation in the embryo. Alternatively, the same sequence elements and factors may underlie both the establishment of methylation-free regions and the retention of nucleosomes in sperm.

### Sperm chromatin organization may drive fine-scale base composition variation in the human genome

Finally, based on the striking relationship between base composition and nucleosome occupancy in sperm, we propose that chromatin organization in the male germline may be an important selective pressure on GC-content evolution in mammalian genomes. By defining the regions at which nucleosomes are retained in the paternal germline, base composition establishes the organization of sperm chromatin and so the regions at which epigenetic information in the form of histone modifications can be transmitted from one generation to the next. It is interesting to speculate, therefore, that a requirement to transmit paternal epigenetic information to the zygote could be an important selective pressure on sequence evolution in mammalian genomes.

## Methods

### Nucleosome retention sites in human sperm

The following measures of nucleosome retention in human sperm were used in this study.

#### Regions of nucleosome retention defined using deep sequencing

We used the genomic positions of retained nucleosomes from four sperm donors as identified using micrococcal nuclease digestion and reported by Hammoud *et al* [5]. In brief, these regions were defined by the USeq package [59], using a 300-bp sliding window along the genome, and represent genomic windows where sequence reads are significantly more from the histone-bound fraction of the genome than from the genomic input control.

#### Nucleosome retention score defined using deep sequencing

The raw unfiltered reads from the nucleosome fraction and genomic input control from four sperm donors were downloaded from Gene Expression Omnibus (GEO) [60]. We filtered reads keeping those matching the genome without mismatches and with an Eland alignment score ≥13 as in [5]. We then shifted the positions of the reads by 75 bp (which is half of the length of the sequenced nucleosome-bound fragments) in the direction of sequencing to transform the data from counts of 5′ and 3′ ends to central nucleosome positions. To account for a possible sequencing bias, we calculated the normalized nucleosome retention score in 150 bp windows genome-wide as the difference between the number of nucleosome reads and the number of genomic input reads within the window and divided by the square root of the sum [5], [59]. Because the nucleosome fraction sample was sequenced to a greater depth compared to the genomic input control, for the calculation of the normalized nucleosome retention score the reads from the nucleosome fraction were randomly sub-sampled to generate a dataset with the same number of reads as the genomic input, as described in [5]. Repetitive windows of the genome were defined using the Duke uniqueness track downloaded from the UCSC browser website [61]. For the analyses of correlation between nucleosome retention and GC-content or nucleosome affinity, 150-bp windows containing non-unique 20mers were removed.

#### Genome-wide nucleosome retention scores defined using microarrays

We also analyzed two additional nucleosome retention scores along the human genome from data generated by a second laboratory [3]. In these experiments the nucleosome-bound regions were isolated by two different experimental methods (micrococcal nuclease digestion (MND), and salt extraction followed by endonuclease digestion (SRD)) from four donors, and identified using genome-wide low-density CGH microarrays. In brief, sperm chromatin was digested with micrococcal nuclease and then centrifuged to separate the histone from the protamine fraction (MND experiment). Alternatively, sperm chromatin was treated with weak salt solutions, digested with two endonucleases and centrifuged to separate the histone and protamine fractions (SRD experiment). In both cases, the histone and the protamine fraction were hybridized to a two-colour CGH array consisting of 44 thousand genic and intergenic probes. The raw hybridization signal intensity data for these two experiments were downloaded from GEO. We normalized the downloaded raw microarray data using MA2C with the “Robust” normalization setting to adjust for dye and probe-sequence bias [62]. We also analyzed data from a third MNase digestion map [4], but found that it shows little agreement with data from the two other studies. The reasons for this are not clear, but may relate to a more extensive digestion of DNA.

### Retention of modified nucleosomes in human sperm

We used the H3K4me3 and H3K27me3 ChIP-Seq data generated by Hammoud *et al* [5]. For each of the two datasets, to control for sequencing biases, we calculated a retention score, based on the binomial distribution, by normalizing against the input control.

### Genomic distribution of regions of nucleosome retention in sperm

We classified genes as overlapping retained nucleosomes at their transcription start sites when one or more start site is within 50 bp of a nucleosome enrichment region defined by sequencing from Hammoud *et al* [5]. Coordinates for all protein-coding and non-coding genes and transcripts were retrieved from Ensembl release 54 [63].

### Nucleosome occupancy in T-cells

We used the nucleosome occupancy data generated by Schones *et al* using MNase digestion and deep sequencing [51]. The positions of the uniquely mapped sequenced reads marking the ends of nucleosomes along the human genome were downloaded from the authors' website. We filtered-out identical reads. As for the sperm nucleosome data, we shifted the positions of the reads in the direction of sequencing to transform the data from positions of fragment ends to central nucleosome positions. We then counted these transformed nucleosome positions along the human genome in 150 bp windows.

### Intrinsic nucleosome binding preferences

Nucleosome binding preferences were predicted using the model of Kaplan *et al* [30], which is trained on the occupancy of chicken nucleosomes on naked yeast DNA. The nucleosome affinity score for human genome version hg18 was downloaded from the authors' website.

### Gene expression data

We retrieved gene expression data for ten tissues (testes, brain, breast, colon, heart, liver, lymph node, skeletal muscle and cerebellum) quantified by Solexa sequencing from Wang *et al* [53]. The expression values from the six samples for cerebellum were averaged. Gene expression was measured in ‘number of sequenced reads per kilobase of exon per million mapped reads’ (RPKM) [64]. We considered genes with ≥0.5 RPKM in a tissue as expressed in that tissue. As tissue-specific genes we defined those with expression above the threshold in one out of ten tissues. We retrieved ubiquitously expressed (housekeeping) human genes from Ramskold *et al* [65].

### mRNA retention in mature sperm

The abundance of mRNA in mature sperm from 13 different fertile donors was measured using Affymetrix gene expression microarrays by Platts *et al* [66]. We downloaded the mRNA detection calls (mRNA present/absent calculated by DChip MBE) for each gene from GEO. Probe to gene mappings were made using Ensembl and probes matching multiple genes were removed. We defined a gene's mRNA as present in mature sperm if at least one probe matching this gene showed expression present in at least 7 out of 13 sperm donors.

### Gene function annotations

Gene ontology (GO) annotations of genes were obtained from Ensembl. Genes coding for developmental transcription factors were defined as genes annotated with the Biological Process term “DNA-dependent regulation of cellular transcription” (GO:0006355) and also with a term that contains the word “development.”

### Predicting nucleosome retention across the human genome

To test the performance of GC-content as a predictor of nucleosome retention throughout the human genome we used all non-repetitive 150 bp windows of the genome. We further excluded windows that had in total less than 5 sequenced reads from the nucleosome and the genomic control datasets, as low-read count windows were also excluded from the nucleosome retention peak finding algorithm used by Hammoud *et al*. This analysis was also performed separately for genic and non-genic windows (here 1 kb upstream of each start site was included in the genic portion of the genome). Receiver operating characteristic (ROC) analysis was used to assess how well we can predict the regions of nucleosome retention enrichment in sperm from GC- and CpG-content. ROC analysis was performed in R using the ROCR package [67]. In brief, all 150 bp windows of the genome were ranked according to decreasing GC (or CpG) count. Going down this ranked list, we then counted the number of windows overlapping regions of nucleosome retention as a fraction of all windows with the same or higher GC-content (true positive rate, y-axis) and the number of windows not overlapping regions of nucleosome retention as a fraction of all windows with lower GC-content (false positive rate, x-axis). If there were no correlation between GC-content and nucleosome retention in sperm, we would expect the ROC curve to be a diagonal line across the plot and the resulting area under the curve to be equal to 0.5. For a perfect predictor, the area under the curve would be equal to 1. In Figure 1, for direct comparison, we also plotted the sensitivity and specificity of CpG islands in predicting 150-bp windows that overlap regions of nucleosome retention in sperm. CpG islands as defined by Gardiner-Garden *et al* [36] were downloaded from the UCSC genome browser database.

### DNase I hypersensitive sites in human embryonic stem cells

We retrieved the locations of DNase I hypersensitive sites for H1 human embryonic stem cells [68] from Ensembl (release 60) and converted the locations to human genome version hg18 using the UCSC LiftOver tool. All sites within 1 kb from any type of annotated gene in Ensembl were removed. We retained 64,217 noncoding ES DNase I hypersensitive sites. Average signals were calculated for 4 kb centered on the middle positions of these sites.

### DNA methylation annotation of CpG islands in human embryonic stem cells

We used the DNA methylation status annotation of CpG islands reported in Straussman *et al* [41]. We considered only CpG islands that were consistently annotated as DNA methylated or unmethylated in both embryonic stem cell lines (I6 and H13) tested by Straussman *et al*. Average signals were calculated for 4 kb regions centered at the middle position of the islands.

## Supporting Information

Figure S1 Correlations between GC-content and nucleosome to protamine normalized probe signal intensity ratio. The two plots show the correlation for the data generated by two different experimental methods of identifying nucleosome enriched regions from Arpanahi *et al*; micrococcal nuclease digestion (MND, A) and salt-extraction followed by restriction digestion (SRD, B). In both cases the probe value corresponds to the ratio of the nucleosome to protamine normalized signal intensity. The GC-content shown corresponds to the window ±147 bp from the microarray probe.
(EPS)

Figure S2 Nucleosomes are preferentially retained in sperm at transcription start sites. (A) Genome-wide distribution of nucleosome retention sites. Nucleosome enrichment regions are classified according to whether they are within 50 bp of a transcription start site (TSS, 9,068 regions), or end site (TES, 718 regions), overlapping other parts of a gene (other genic, 7,785) or other parts of the genome (non-genic, 7,549). The coordinates of regions of nucleosome retention defined by sequencing were retrieved from Hammoud *et al*. (B) The plot shows the percentage of transcription start and end sites that are within 50 bp of a nucleosome retention region. The total number of human (Ensembl) transcripts considered here is 52,312. Error bars indicate 95% confidence intervals.
(EPS)

Figure S3 Nucleosome retention at transcription start sites correlates with the expression breadth of a gene rather than the expression level in the germline. (A) There is a positive trend in the proportion of genes that have retained nucleosomes at the start site and the expression level of the gene in testes (1,589 genes per bin, χ^2^ -test for trend in proportions = 626.1, p-value<2.2×10^−16^). (B) However, there is also a strong positive correlation between expression breadth (the number of different tissues in which a gene is expressed) and nucleosome retention at the start site (χ^2^ -test for trend in proportions = 1,303.7, p-value<2.2×10^−16^). The total number of genes (and the number of genes with nucleosomes retained at the start) in each bin are as follows: 0-tissues = 792 (125), 1-tissue = 1472 (313), 2-tissues = 835 (284), 3-tissues = 742 (316), 4-tissues = 653 (264), 5-tissues = 562 (251), 6-tissues = 617 (287), 7-tissues = 657 (324), 8-tissues = 817 (433), 9-tissues = 1248 (711), 10-tissues = 7502 (4555). (C) The correlation between nucleosome retention at the start and expression level is largely explained by expression breadth. Here, we test the correlation between expression level and nucleosome retention at the start of genes, when controlling for expression breadth. In C, each panel corresponds to the genes in each expression breadth shown in panel B. Genes are sorted according to expression level and split in ten equally sized bins. The x-axis shows the average expression level of the genes in each bin. The y-axis shows the percentage of genes in a bin that have a start site within 50 bp from a region of nucleosome retention in sperm. From this figure it is also apparent that when controlling for expression level, the expression breadth of a gene correlates with the likelihood of nucleosome retention at the gene start (e.g. compare high expression level bins for genes expressed in one versus ten tissues).
(EPS)

Figure S4 Relationship between nucleosome retention at transcription start sites and the expression level or breadth of mRNAs detected as present in the mature sperm of at least seven out of thirteen healthy sperm donors. (A) The percentage of genes that retain nucleosomes at their start increases with gene expression level (522 genes per bin, χ^2^ -test for trend in proportions = 158.9, p-value<2.2×10^−16^). (B) Expression breadth shows strong correlation with nucleosome retention at the gene start (χ^2^ -test for trend in proportions = 512.8, p-value<2.2×10^−16^). The number of genes per bin are; 166 (0 tissues), 632 (1 tissue), 293 (2 tissues), 304 (3 tissues), 273 (4 tissues), 233 (5 tissues), 283 (6 tissues), 318 (7 tissues), 419 (8 tissues), 763 (9 tissues), 5,222 (10 tissues). (C) When considering genes expressed in one tissue only, i.e. tissue-specific genes, the trend between nucleosome retention and expression level in the germline is greatly reduced (135 genes per bin, χ^2^ -test for trend in proportions = 1.158, p-value = 0.28). (D) When considering genes expressed in all tissues, i.e. housekeeping genes, the trend between nucleosome retention and expression level in the germline is also greatly reduced (522 genes per bin, χ^2^ -test for trend in proportions = 0.041, p-value = 0.84). All genes with testes expression >0 and with present mRNA in at least seven out of thirteen healthy sperm donors are considered here (N = 5,222). Genes are considered to be expressed in a tissue when their expression level is > = 0.5 RPKM. Genes are ranked according to increasing expression level in testes and split in bins with the same number of genes. The x-axis shows the average expression level of the genes in each bin. The y-axis shows the percentage of genes in a bin that have a start site within 50 bp from a region of nucleosome retention in sperm.
(EPS)

Figure S5 Sperm H3K4me3, H3K27me3 and H2A.Z retention at the start sites of housekeeping, tissue-specific and developmental regulatory genes. (A–C) Retention of H3K4me3 at the start sites of the three gene classes is similar to nucleosome retention, shown in main Figure 3E–3G. (D–F) Retention of H3K27me3 appears to be enriched around the start sites of developmental regulators, as previously noted by Hammoud *et al* and Brykczynska *et al*. (G–I) Histone variant H2A.Z shows no enriched at gene start sites. This agrees with the observation that this histone variant is only enriched in pericentric heterochromatin.
(EPS)

Figure S6 Correlations between sperm nucleosome retention and GC- or CpG-content. Each point shows the correlation for all unique 150-bp windows in the human genome with the same number of G+C nucleotides (A) or CpG dinucleotides (B). GC-content correlates robustly with nucleosome retention over a wide range of window CpG-contents, whereas CpG content only correlates strongly with retention at high GC-contents. The analysis was carried out on windows with at least 5 reads from the histone and the input control dataset, as in Figure 1. Correlation values are Pearson correlation coefficients and error bars represent 95% confidence intervals. 95% of all windows fall within the data ranges shown here.
(EPS)

## References

[1] Puwaravutipanich T, Panyim S (1975). The nuclear basic proteins of human testes and ejaculated spermatozoa.. Exp Cell Res.

[2] Ward WS, Coffey DS (1991). DNA packaging and organization in mammalian spermatozoa: comparison with somatic cells.. Biol Reprod.

[3] Arpanahi A, Brinkworth M, Iles D, Krawetz SA, Paradowska A (2009). Endonuclease-sensitive regions of human spermatozoal chromatin are highly enriched in promoter and CTCF binding sequences.. Genome Res.

[4] Brykczynska U, Hisano M, Erkek S, Ramos L, Oakeley EJ (2010). Repressive and active histone methylation mark distinct promoters in human and mouse spermatozoa.. Nat Struct Mol Biol.

[5] Hammoud SS, Nix DA, Zhang H, Purwar J, Carrell DT (2009). Distinctive chromatin in human sperm packages genes for embryo development.. Nature.

[6] Pogany GC, Corzett M, Weston S, Balhorn R (1981). DNA and protein content of mouse sperm. Implications regarding sperm chromatin structure.. Exp Cell Res.

[7] de Yebra L, Ballesca JL, Vanrell JA, Bassas L, Oliva R (1993). Complete selective absence of protamine P2 in humans.. J Biol Chem.

[8] Brewer L, Corzett M, Lau EY, Balhorn R (2003). Dynamics of protamine 1 binding to single DNA molecules.. J Biol Chem.

[9] Ward WS (2009). Function of sperm chromatin structural elements in fertilization and development.. Mol Hum Reprod.

[10] Cho C, Willis WD, Goulding EH, Jung-Ha H, Choi YC (2001). Haploinsufficiency of protamine-1 or -2 causes infertility in mice.. Nat Genet.

[11] Cho C, Jung-Ha H, Willis WD, Goulding EH, Stein P (2003). Protamine 2 deficiency leads to sperm DNA damage and embryo death in mice.. Biol Reprod.

[12] Haueter S, Kawasumi M, Asner I, Brykczynska U, Cinelli P (2010). Genetic vasectomy-overexpression of Prm1-EGFP fusion protein in elongating spermatids causes dominant male sterility in mice.. Genesis.

[13] Gardiner-Garden M, Ballesteros M, Gordon M, Tam PP (1998). Histone- and protamine-DNA association: conservation of different patterns within the beta-globin domain in human sperm.. Mol Cell Biol.

[14] Wykes SM, Krawetz SA (2003). The structural organization of sperm chromatin.. J Biol Chem.

[15] Gatewood JM, Cook GR, Balhorn R, Bradbury EM, Schmid CW (1987). Sequence-specific packaging of DNA in human sperm chromatin.. Science.

[16] van der Heijden GW, Ramos L, Baart EB, van den Berg IM, Derijck AA (2008). Sperm-derived histones contribute to zygotic chromatin in humans.. BMC Dev Biol.

[17] van der Heijden GW, Derijck AA, Ramos L, Giele M, van der Vlag J (2006). Transmission of modified nucleosomes from the mouse male germline to the zygote and subsequent remodeling of paternal chromatin.. Dev Biol.

[18] Puschendorf M, Terranova R, Boutsma E, Mao X, Isono K (2008). PRC1 and Suv39h specify parental asymmetry at constitutive heterochromatin in early mouse embryos.. Nat Genet.

[19] Carone BR, Fauquier L, Habib N, Shea JM, Hart CE Paternally induced transgenerational environmental reprogramming of metabolic gene expression in mammals.. Cell.

[20] Ng SF, Lin RC, Laybutt DR, Barres R, Owens JA (2010). Chronic high-fat diet in fathers programs beta-cell dysfunction in female rat offspring.. Nature.

[21] Youngson NA, Whitelaw E (2008). Transgenerational epigenetic effects.. Annu Rev Genomics Hum Genet.

[22] Kornberg RD, Stryer L (1988). Statistical distributions of nucleosomes: nonrandom locations by a stochastic mechanism.. Nucleic Acids Res.

[23] Mavrich TN, Ioshikhes IP, Venters BJ, Jiang C, Tomsho LP (2008). A barrier nucleosome model for statistical positioning of nucleosomes throughout the yeast genome.. Genome Res.

[24] Weiner A, Hughes A, Yassour M, Rando OJ, Friedman N (2009). High-resolution nucleosome mapping reveals transcription-dependent promoter packaging.. Genome Res.

[25] Zhang Y, Moqtaderi Z, Rattner BP, Euskirchen G, Snyder M (2009). Intrinsic histone-DNA interactions are not the major determinant of nucleosome positions in vivo.. Nat Struct Mol Biol.

[26] Drew HR, Travers AA (1985). DNA bending and its relation to nucleosome positioning.. J Mol Biol.

[27] Lowary PT, Widom J (1998). New DNA sequence rules for high affinity binding to histone octamer and sequence-directed nucleosome positioning.. J Mol Biol.

[28] Segal E, Fondufe-Mittendorf Y, Chen L, Thastrom A, Field Y (2006). A genomic code for nucleosome positioning.. Nature.

[29] Field Y, Kaplan N, Fondufe-Mittendorf Y, Moore IK, Sharon E (2008). Distinct modes of regulation by chromatin encoded through nucleosome positioning signals.. PLoS Comput Biol.

[30] Kaplan N, Moore IK, Fondufe-Mittendorf Y, Gossett AJ, Tillo D (2009). The DNA-encoded nucleosome organization of a eukaryotic genome.. Nature.

[31] Tillo D, Hughes TR (2009). G+C content dominates intrinsic nucleosome occupancy.. BMC Bioinformatics.

[32] Chung HR, Vingron M (2009). Sequence-dependent nucleosome positioning.. J Mol Biol.

[33] Vinogradov AE (2005). Noncoding DNA, isochores and gene expression: nucleosome formation potential.. Nucleic Acids Res.

[34] Tillo D, Kaplan N, Moore IK, Fondufe-Mittendorf Y, Gossett AJ (2010). High nucleosome occupancy is encoded at human regulatory sequences.. PLoS ONE.

[35] Ramirez-Carrozzi VR, Braas D, Bhatt DM, Cheng CS, Hong C (2009). A unifying model for the selective regulation of inducible transcription by CpG islands and nucleosome remodeling.. Cell.

[36] Gardiner-Garden M, Frommer M (1987). CpG islands in vertebrate genomes.. J Mol Biol.

[37] Schug J, Schuller WP, Kappen C, Salbaum JM, Bucan M (2005). Promoter features related to tissue specificity as measured by Shannon entropy.. Genome Biol.

[38] Tanay A, O'Donnell AH, Damelin M, Bestor TH (2007). Hyperconserved CpG domains underlie Polycomb-binding sites.. Proc Natl Acad Sci U S A.

[39] Mohn F, Weber M, Rebhan M, Roloff TC, Richter J (2008). Lineage-specific polycomb targets and de novo DNA methylation define restriction and potential of neuronal progenitors.. Mol Cell.

[40] Bird A, Taggart M, Frommer M, Miller OJ, Macleod D (1985). A fraction of the mouse genome that is derived from islands of nonmethylated, CpG-rich DNA.. Cell.

[41] Straussman R, Nejman D, Roberts D, Steinfeld I, Blum B (2009). Developmental programming of CpG island methylation profiles in the human genome.. Nat Struct Mol Biol.

[42] Weber M, Hellmann I, Stadler MB, Ramos L, Paabo S (2007). Distribution, silencing potential and evolutionary impact of promoter DNA methylation in the human genome.. Nat Genet.

[43] Frank D, Keshet I, Shani M, Levine A, Razin A (1991). Demethylation of CpG islands in embryonic cells.. Nature.

[44] Chodavarapu RK, Feng S, Bernatavichute YV, Chen PY, Stroud H (2010). Relationship between nucleosome positioning and DNA methylation.. Nature.

[45] Kafri T, Ariel M, Brandeis M, Shemer R, Urven L (1992). Developmental pattern of gene-specific DNA methylation in the mouse embryo and germ line.. Genes Dev.

[46] Brandeis M, Frank D, Keshet I, Siegfried Z, Mendelsohn M (1994). Sp1 elements protect a CpG island from de novo methylation.. Nature.

[47] Macleod D, Charlton J, Mullins J, Bird AP (1994). Sp1 sites in the mouse aprt gene promoter are required to prevent methylation of the CpG island.. Genes Dev.

[48] Ooi SK, Qiu C, Bernstein E, Li K, Jia D (2007). DNMT3L connects unmethylated lysine 4 of histone H3 to de novo methylation of DNA.. Nature.

[49] Lander ES, Linton LM, Birren B, Nusbaum C, Zody MC (2001). Initial sequencing and analysis of the human genome.. Nature.

[50] Dohm JC, Lottaz C, Borodina T, Himmelbauer H (2008). Substantial biases in ultra-short read data sets from high-throughput DNA sequencing.. Nucleic Acids Res.

[51] Schones DE, Cui K, Cuddapah S, Roh TY, Barski A (2008). Dynamic regulation of nucleosome positioning in the human genome.. Cell.

[52] Monesi V (1965). Differential rate of ribonucleic acid synthesis in the autosomes and sex chromosomes during male meiosis in the mouse.. Chromosoma.

[53] Wang ET, Sandberg R, Luo S, Khrebtukova I, Zhang L (2008). Alternative isoform regulation in human tissue transcriptomes.. Nature.

[54] Lidor Nili E, Field Y, Lubling Y, Widom J, Oren M (2010). p53 binds preferentially to genomic regions with high DNA-encoded nucleosome occupancy.. Genome Res.

[55] Hajkova P (2010). Epigenetic reprogramming–taking a lesson from the embryo.. Curr Opin Cell Biol.

[56] Thomson JP, Skene PJ, Selfridge J, Clouaire T, Guy J (2010). CpG islands influence chromatin structure via the CpG-binding protein Cfp1.. Nature.

[57] Hajkova P, Ancelin K, Waldmann T, Lacoste N, Lange UC (2008). Chromatin dynamics during epigenetic reprogramming in the mouse germ line.. Nature.

[58] Tirosh I, Barkai N (2008). Two strategies for gene regulation by promoter nucleosomes.. Genome Res.

[59] Nix DA, Courdy SJ, Boucher KM (2008). Empirical methods for controlling false positives and estimating confidence in ChIP-Seq peaks.. BMC Bioinformatics.

[60] Barrett T, Troup DB, Wilhite SE, Ledoux P, Rudnev D (2009). NCBI GEO: archive for high-throughput functional genomic data.. Nucleic Acids Res.

[61] Rhead B, Karolchik D, Kuhn RM, Hinrichs AS, Zweig AS (2010). The UCSC Genome Browser database: update 2010.. Nucleic Acids Res.

[62] Song JS, Johnson WE, Zhu X, Zhang X, Li W (2007). Model-based analysis of two-color arrays (MA2C).. Genome Biol.

[63] Hubbard TJ, Aken BL, Ayling S, Ballester B, Beal K (2009). Ensembl 2009.. Nucleic Acids Res.

[64] Mortazavi A, Williams BA, McCue K, Schaeffer L, Wold B (2008). Mapping and quantifying mammalian transcriptomes by RNA-Seq.. Nat Methods.

[65] Ramskold D, Wang ET, Burge CB, Sandberg R (2009). An abundance of ubiquitously expressed genes revealed by tissue transcriptome sequence data.. PLoS Comput Biol.

[66] Platts AE, Dix DJ, Chemes HE, Thompson KE, Goodrich R (2007). Success and failure in human spermatogenesis as revealed by teratozoospermic RNAs.. Hum Mol Genet.

[67] Sing T, Sander O, Beerenwinkel N, Lengauer T (2005). ROCR: visualizing classifier performance in R.. Bioinformatics.

[68] Sabo PJ, Kuehn MS, Thurman R, Johnson BE, Johnson EM (2006). Genome-scale mapping of DNase I sensitivity in vivo using tiling DNA microarrays.. Nat Methods.

